# Editorial: New strategies to tackle chemoresistance in cancer

**DOI:** 10.3389/fonc.2022.1062921

**Published:** 2022-10-25

**Authors:** Carmela De Marco, Maura Sonego, Anna Martina Battaglia, Giuseppe Viglietto, Flavia Biamonte

**Affiliations:** ^1^ Department of Experimental and Clinical Medicine, “Magna Graecia” University, Catanzaro, Italy; ^2^ Center of Interdepartmental Services (CIS), “Magna Graecia” University, Catanzaro, Italy; ^3^ Division of Molecular Oncology, Centro di Riferimento Oncologico di Aviano (CRO), Istituto di Ricovero e Cura a Carattere Scientifico (IRCCS), National Cancer Institute, Aviano, Italy

**Keywords:** chemoresistance, cancer, cell cycle inhibitors, immune escape, ferroptosis, miR-506-3p, FBXW7

In recent years, the spectrum of therapeutic strategies for cancer patients has been considerably expanded. However, successful cancer therapy remains a major challenge as both inherent genetic characteristics and adaptive mechanisms resulting from drug exposure may lead to resistance to therapy. Innate characteristics of drug-resistant cells include enhanced DNA repair activity, and defects in apoptotic and cell cycle-related pathways. Moreover, depending on the therapeutic approach, cancer cells as well as cells residing within the tumor microenvironment (TME) may employ strategies such as metabolic rewiring and immune evasion to survive the stress caused by drug exposure (1, 2).

With our Research Topic “*New Strategies to Tackle Chemoresistance in Cancer*” we aimed to initiate generating a collaborative discussion on new ways to overcome drug resistance and potentially improve cancer patient outcome. In total, we collected 5 articles (3 original research articles, 1 review, and 1 mini review).

Epithelial ovarian cancer (EOC) has one of the highest deaths rate (59%) among female tumor (3–5). Therefore, improving survival and response to therapy is still a major challenge. Depending on the histological type and staging of the tumor, modern approaches include primary debulking surgery followed by platinum-based adjuvant therapy, angiogenesis inhibitors, poly ADP-ribose polymerase inhibitors (PARPi), and immunotherapies. However, despite a good response rate to first-line therapy, the vast majority of EOC patients relapse (6). In their original research article, Bagnoli et al. demonstrated that the ectopic expression of miR-506-3p in platinum-resistant cell lines, decreases the expression of the early sensor of DNA damage RAD17, thereby attenuating the ability of EOC cells to recognize DNA damage and abrogating the G2/M cell cycle checkpoint. The consequent delay in the G2/M cell cycle arrest in response to DNA damage allows severely DNA-damaged cells to enter mitosis and leads to overall sensitization to platinum treatment. Furthermore, miR-506-3p expression possibly by inhibiting RAD17 expression impairs the cell cycle checkpoint kinases Chk1 and Wee1 inhibitors by enhancing their activity.

Three selective CDK4/6 small inhibitors (CDKi) (Palbociclib, Ribociclib, and Abemaciclib) have drastically changed the therapeutic approach for hormone-positive (HR+) advanced breast cancer (BC) patients (7, 8). However, since their approval, clinical evidence has demonstrated that about 30% of BC is intrinsically resistant to CDK4/6 inhibitors and that prolonged treatment eventually leads to acquired resistance in many patients. In their minireview, Rampioni Vinciguerra et al. recapitulate that cancer cells adopt different strategies to resist CDK4/6 inhibitors, including both cell cycle specific and nonspecific mechanisms. Cell cycle-specific mechanisms encompass the incomplete inactivation of CDK4/6, the capability of cyclin E1/E2-CDK2 complex to initiate the phosphorylation of the retinoblastoma protein (Rb), and the direct inactivation of Rb, whereas cell cycle-nonspecific mechanisms rely on different pathways, especially like Receptor Tyrosine Kinases (RTK) and PI3K-AKT-mTOR axis. Combining CDK-i with other compounds appears a reasonable and necessary strategy to improve their efficacy and prevent or revert a resistant phenotype. When CDK-i is administered in combination with conventional chemotherapy, its efficacy strongly depends on the treatment schedule, because only the administration of CDK-i after the chemotherapeutic drug can prevent neoplastic cell recovery and lead to a synergistic effect. Otherwise, cell-cycle blockade induced by CDK-i may even protect tumor cells from the cytotoxic effects of chemotherapy. The combination of CDK-i with other targeted therapies is still an emerging but rapidly expanding field. Similarly, promising evidence supports the possibility that CDK-i may enhance the effects of immunotherapy with checkpoint inhibitors.

In EOC, ferroptosis inducers such as erastin and sulfasalazine are showing promising antitumor effects but the existence of still undefined genetic and metabolic determinants of susceptibility has so far limited their application *in vivo* (9, 10). In their article, Battaglia et al. provided evidence that baseline and the induced intracellular free iron level are significant determinants of erastin-mediated ferroptosis sensitivity. In particular, they demonstrated that erastin induces further intracellular iron accumulation through ferritinophagy in HEY cells that have high baseline free iron levels, culminating in mitochondrial dysfunction. Conversely, COV318 cells, with low baseline intracellular labile iron pool, appeared to be resistant to erastin treatment. Interestingly, the use of the ferric iron compound ferlixit, normally used to treat patients with anemia, sensitizes COV318 cells to erastin through ferritinophagy-independent intracellular iron accumulation and mitochondrial dysfunction.

In their review, Shen et al. described the role of FBXW7, a member of the F-box protein family that plays an important role in controlling various cellular processes (e.g., cell proliferation and death, metabolism, invasion and metastasis, immune escape, and genomic instability). They propose FBXW7 as a therapeutic target of miRNA, wake-up therapy, and chronotherapy, the latter being an attractive strategy in which anticancer drugs are administered with optimal timing according to circadian rhythms of anticancer effects and adverse effects on normal cells.

Jiang et al. highlighted the importance of TME in the immune escape of bladder cancer (BLCA). Recent breakthroughs in cancer biology have shown that tumors promote the formation of a highly immunosuppressive microenvironment that prevents an effective immune response against the tumor through multiple mechanisms. Since immunotherapy is one of the most promising treatment strategies, reconditioning the tumor microenvironment and restoring a competent immune response is critical for the optimal efficacy of cancer immunotherapy. Jiang et al. performed an integrated analysis of BLCA samples collected from The Cancer Genome Atlas (TCGA) to classify bladder tumors into two clusters based on immune score: high immune score (ImmuneScoreH) and low immune score (ImmuneScoreL), respectively. The two clusters differ significantly in both cell composition and overall survival. In addition, analysis of the differentially expressed genes (DEGs) in the two clusters helped Jiang et al. to establish a prognostic gene signature based on the expression of CD96 and IBSP.

Altogether, the articles collected in this Research Topic provide a series of insightful sets of data to better understand the molecular processes involved in the development of drug resistance in cancer and pave the way for more interdisciplinary work aimed at improving the treatment of cancer (**Figure 1**).

**Figure 1:**
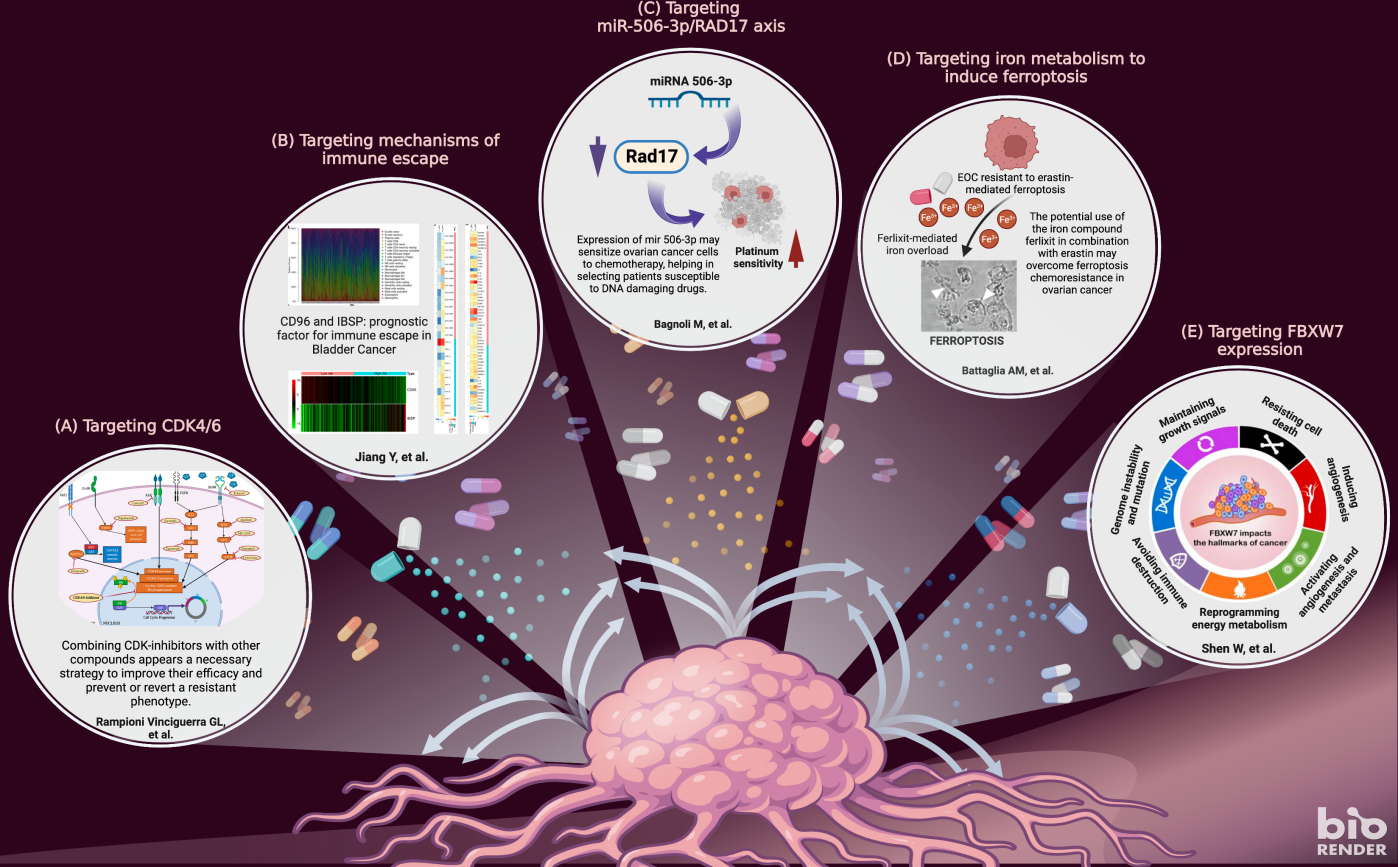
The figure highlights the topics covered in the research articles included in this special issue. The articles provide a detailed overview of five important topics in tackling chemoresistance in cancer: targeting cyclin-dependent kinases **(A)**; targeting immune escape **(B)**; targeting miR-506/RAD17 axis **(C)**; targeting ferroptosis **(D)**; protein FBXW7 factor **(E)**. Adapted from “New strategies for treating cancer” by BioRender.com (2022). Retrieved from https://app.biorender.com/biorender-templates.

## Author contributions

All authors listed have made a substantial, direct and intellectual contribution to the work, and approved it for publication.

## Conflict of interest

The authors declare that the research was conducted in the absence of any commercial or financial relationships that could be construed as a potential conflict of interest.

## Publisher’s note

All claims expressed in this article are solely those of the authors and do not necessarily represent those of their affiliated organizations, or those of the publisher, the editors and the reviewers. Any product that may be evaluated in this article, or claim that may be made by its manufacturer, is not guaranteed or endorsed by the publisher.
